# Editorial: Hold the salt: dietary sodium's effect on cardiovascular and kidney diseases

**DOI:** 10.3389/fnut.2024.1533076

**Published:** 2024-12-18

**Authors:** Marcelo Perim Baldo, Maria do Carmo Serrano, Ashley Pitzer Mutchler, Youngseung Lee

**Affiliations:** ^1^Cardiovascular Research Center (CPC/LAMICC), Montes Claros State University, Montes Claros, Brazil; ^2^Instituto Nacional de Investigação Agrária e Veterinária (INIAV, I.P.), Oeiras, Portugal; ^3^LEAF-Linking Landscape, Environment, Agriculture and Food Research Unit, Associated Laboratory TERRA, Intituto Superior de Agronomia, Universidade de Lisboa, Lisbon, Portugal; ^4^Division of Clinical Pharmacology, Department of Medicine, Vanderbilt University Medical Center, Nashville, TN, United States; ^5^Department of Food Science and Nutrition, Dankook University, Cheonan-si, Republic of Korea

**Keywords:** salt intake, cardiovascular disease, kidney disease, epidemiology, public health

Salt, or sodium chloride, has played a pivotal role in human history as a vital nutrient and as a commodity that shaped civilizations. Its importance dates back to prehistoric times, with archaeological evidence suggesting that early humans sought salt-rich environments to support their dietary needs. Salt was not only essential for survival but also became a key factor in the development of trade routes, influencing the rise and fall of empires. Ancient civilizations, such as the Egyptians, used salt for food preservation and mummification, showcasing its wide range of applications. As society evolved, salt became more than a nutritional element—it was central to religious rituals, cultural practices, and even currency systems (1).

The relationship between salt and health, however, has been more complex. Historically, salt was recognized as necessary for maintaining bodily functions, particularly in regulating fluid balance and nerve transmission. However, excessive salt consumption has been linked to adverse health outcomes, particularly cardiovascular and kidney diseases, which remain a global concern today. Numerous studies have established a strong relationship between high sodium intake and adverse cardiovascular outcomes, and consequently, public health guidelines recommend limiting sodium intake to reduce these risks. A meta-analysis demonstrated that excessive sodium intake is associated with elevated blood pressure, a well-known risk factor for cardiovascular diseases (CVD) such as myocardial infarction and stroke (2). The Global Burden of Disease Study found that high sodium consumption was responsible for 3 million deaths and 70 million disability-adjusted life years (DALYs) globally, highlighting its significant contribution to CVD (3). Furthermore, clinical studies such as the Dietary Approach to Stop Hypertension (DASH) study have shown that reducing salt intake can significantly lower blood pressure and decrease the risk of cardiovascular events (4). This body of evidence has led global health organizations, including the World Health Organization (WHO), to recommend limiting sodium intake to < 2 g daily to mitigate cardiovascular risk.

In addition to cardiovascular risk, high sodium intake has detrimental effects on kidney function. Chronic high salt consumption is linked to the progression of chronic kidney disease (CKD) and an increased risk of end-stage renal disease (ESRD) (5). Studies highlighted that high dietary salt exacerbates proteinuria and accelerates the decline in kidney function among patients with hypertension and CKD. Moreover, excessive sodium intake can induce glomerular hyperfiltration and promote kidney damage over time, particularly in individuals with pre-existing renal conditions (6–8). These findings underscore the importance of salt reduction not only for cardiovascular health but also for preventing and managing kidney disease.

Research into the effects of salt on the cardiovascular and kidney systems is essential for addressing rising global health challenges like hypertension, heart diseases, and CKD. Despite widespread awareness, gaps remain in our understanding of the specific mechanisms by which excess salt harms these systems. By offering fresh insights, our Research Topic fills this critical need, presenting new findings that highlight the nuanced impact of salt at clinical, epidemiological, and molecular levels.

A study on the North Indian population (Kaur et al.) found that sodium and salt consumption exceed recommended levels, while potassium intake remains below ideal. This imbalance is particularly concerning among individuals with CKD, suggesting the need for targeted dietary policies to mitigate CKD progression and improve overall public health outcomes. In a related study, high salt intake and over hydration among non-dialysis CKD patients were linked to an increased risk of cardiac structural and functional impairments (Duan et al.). An experimental study on rodents (Siddiqui et al.) revealed that diets high in fructose and salt led to cardiorenal dysfunctions. However, the chronic inhibition of the renin-angiotensin system (RAS) improved both cardiac and renal histopathological outcomes.

In the realm of the heart disease, a study on rheumatic heart disease (RHD; Zhang et al.) showed a decline in RHD-related mortality and DALYs over 30 years, with high systolic blood pressure identified as a key risk factor. This underscores the need for innovative, personalized interventions to reduce sodium intake and improve overall nutrient consumption, especially in light of cardiovascular and renal health goals. Moreover, research on hospitalized patients with dysnatremia (Liang et al.) found that sodium fluctuations are directly linked to increased mortality, emphasizing the importance of strict monitoring of sodium levels in clinical settings.

Investigations into potassium intake (Yuan et al.) demonstrated that higher potassium consumption is associated with a reduced risk of albuminuria, a marker of kidney disease. Another study (Xu et al.) revealed that the urinary sodium/potassium ratio (Na/K) correlates strongly with hypertension and blood pressure indices. This suggests that the Na/K ratio can serve as an effective tool for monitoring dietary sodium reduction and potassium increase. Further research into sodium-induced hypertension in the Black population of Sub-Saharan Africa (Masenga et al.) uncovered significant sex differences in sodium sensitivity. Women, in particular, exhibited a more pronounced disruption of the vascular endothelial glycocalyx following acute salt load, highlighting the importance of sex-specific intervention strategies for cardiovascular disease prevention.

Efforts to reduce sodium in food products are also advancing. For example, high-pressure processing in processed meats was shown to enhance saltiness perception and improve sensory acceptability without compromising taste (Bolumar et al.). Similarly, enzyme treatments in vegetable soups were found to increase saltiness and umami intensity, allowing for a 20% reduction in salt while maintaining clean-label requirements (Sakai et al.). Additionally, research on microencapsulated ingredients from aromatic plants and spices demonstrated their potential to significantly reduce salt content in sauces such as mayonnaise, mustard, and ketchup, permitting a reduction of up to 50%, without sacrificing taste (Serrano et al.). Finally, Kugler et al. study contributes to nutritional epidemiology by providing an extensive analysis of the salt content in bread sold in Slovenia, highlighting significant differences across bread types and retail environments. It underscores the need to include smaller bakeries in salt reduction efforts and reveals that, despite a modest decrease in bread salt content over a decade, WHO targets remain unmet, suggesting further reformulation strategies are necessary.

In light of these findings, we are undoubtedly at a pivotal moment in addressing the global health impacts of excessive sodium intake. The extensively body of evidence, including the studies presented in this Research Topic, leaves no room for hesitation: innovative, science-driven strategies to reduce sodium consumption are not just necessary—they are urgent. From targeted public health policies to advancements in food technologies, the solutions are within our grasp. However, realizing these solutions will require a unified effort from researchers, policymakers, and the food industry. As we move forward, the challenge lies in transforming this knowledge into concrete actions that prioritize both public health and the sustainability of our food systems.
